# A Phase II Study of Docetaxel, Cisplatin and 5- Fluorouracil (TPF) In Patients with Locally Advanced Head and Neck Carcinomas

**Published:** 2011-03-01

**Authors:** M Ansari, S Omidvari, A Mosalaei, N Ahmadloo, M A Mosleh-Shirazi, M Mohammadianpanah

**Affiliations:** 1 Department of Radiation Oncology, Nemazee Hospital, Shiraz University of Medical Sciences, Shiraz, Iran

**Keywords:** Docetaxel, Cisplatin, 5-Fluorouracil, Chemotherapy, Head and neck, Carcinoma

## Abstract

**Background:**

The combination of cisplatin and 5-fluorouracil (PF) is currently considered a standard and effective regimen for the treatment of advanced head and neck carcinomas. The aim of this study was to evaluate the efficacy and safety of docetaxel, cisplatin and 5-fluorouracil (TPF) in patients with unresectable head and neck carcinomas.

**Methods:**

Forty-six patients with previously untreated non-metastatic stage IV head and neck carcinomas were enrolled. All patients received three cycles of induction chemotherapy with docetaxel (75 mg/m(2)), cisplatin (40 mg/m(2)) (days 1-2), and 5-FU (500 mg/m(2), days 1-3), repeated every 21 days. Following induction chemotherapy, all patients underwent concurrent chemoradiotherapy using weekly cisplatin (30 mg/m(2)) and a median total dose of 70 Gy was delivered. Clinical response rate and toxicity were the primary and secondary end-points of the study.

**Results:**

There were 31 men and 15 women. All patients had non-metastatic stage IV (T2-3N2-3 or T4N0-3) of disease. Overall and complete response rates were 74% and 24% respectively. Advanced T4 classification was associated with poorer response rate (p value=0.042). The major (grade 3-4) treatment-related toxicities were myelosuppression (78%), anorexia (13%), diarrhea (7%), emesis (11%) and stomatitis/pharyngitis (24%).

**Conclusion:**

In comparison with the data of historical published trials of the PF regimen, the TPF regimen was more effective. However, the TPF regimen appears to be associated with a higher incidence of major toxicities. Therefore, our limited findings support the TPF regimen as an alternative chemotherapeutic regimen for advanced head and neck carcinomas.

## Introduction

The squamous cell carcinomas of head and neck are the most frequent malignancies in this region.[1] The prognosis of these tumors is directly dependent on their presenting stage. The early stage head and neck carcinomas have favorable prognosis, with 70% to 90% five-year survival rate after a standard treatment (surgery, radiotherapy or a combination of both), but unfortunately about two thirds of patients have locally advanced (stage III and IV) disease.[2] The prognosis for this group of patients is poor.[3] The treatment of choice for these patients was radiotherapy alone with 5-year survival rate of less than 20%.[4][5] The replacement of concurrent chemoradiotherapy [6][7] instead of radiotherapy alone is widely accepted, but induction chemotherapy has also been associated with a survival benefit and could be a valuable treatment option. [5][8][9] The combination of cisplatin and 5- fluorouracil (5-FU), which is called the PF regimen, is the standard regimen for treatment of locally advanced carcinomas of the head and neck region and is effective in these tumors. There were several new agents introduced in the last decade of the 20(th) century,in which the taxanes have shown great effectiveness in the treatment of head and neck carcinomas by addition of docetaxel to cisplatin and 5-FU (the TPF regimen); some phase II studies have revealed that it is more effective than the standard PF regimen. [10][11][12][13][14] Therefore, we arranged a phase II study of the combination of docetaxel with cisplatin and 5-FU in a patient population consisting of locally advanced, nonmetastatic head and neck carcinomas.

## Materials and Methods

This phase II study enrolled patients with newly diagnosed locally advanced (T3-4, and/or N2-3, M0) non-metastatic head and neck carcinomas. From April 2001 to November 2005, 50 Patients with locally advanced head and neck carcinomas were qualified for enrollment in this study. Tumors of the nasopharyngeal origin were excluded. All patients had pathology proof for their malignancies and they were locally advanced, non-metastatic, unresectable with WHO performance status of <2. All of them were between 25 and 75 years old and none were treated previously. They had adequate bone marrow, liver and renal function. The exclusion criteria were previous treatment (chemotherapy or radiotherapy), any metastatic disease or another active malignancy, any radical surgery previously in head and neck region for malignancy, and active co-morbid disease such as uncontrolled diabetes or hypertension or chronic obstructive pulmonary disease requiring hospitalization during the previous year. The trial was approved by the local university Ethics Committee and all patients signed a written informed consent before therapy.

Fifty patients were enrolled in the study but four of them were excluded due to poor cooperation. Surgery was performed four weeks after completion of chemoradiation. The clinical response rate and toxicity were the primary and secondary end-points of this study.

The patients received three cycles of the TPF regimen (docetaxel, 75 mg per square meter of bodysurface area; cisplatin, 40 mg per square meter for the first two consecutive days of each cycle and bolus 5- FU, 500 mg per square meter as bolus for the first three consecutive days of each cycle) as induction chemotherapy, and after three to four weeks following the last (third) cycle of induction chemotherapy, they were assigned to receive concurrent chemoradiation. They received weekly cisplatin (30 mg per square meter per week) during the course of a conventional radiotherapy course. The radiotherapy dose to primary disease and any other gross bulky tumor in the neck was between 65 and 70 Gy, which was administered with definitive and curative intent using 1.8 Gy dose per fraction per day, for 5 days per week. The uninvolved regions received 45-50 Gy, the operation was considered 6 to 8 weeks after the completion of chemoradiotherapy for the patients who had an advanced nodal disease (N2 or N3) on presentation or those who had residual disease after the course of treatment.

Before starting therapy, pretreatment evaluation included a complete patient history and physical examination, computed tomography (CT) of the neck, magnetic resonance imaging (MRI) of the primary site, and chest x-ray. The 6th edition of American Joint Committee on Cancer (AJCC), TNM 2002 staging system was used for staging disease. Complete laboratory tests included a complete blood count (CBC), blood serum electrolytes, creatinine, blood urea nitrogen, liver function tests (LFT), and renal function tests (RFT).

The response to treatment was also assessed the same way after induction chemotherapy and 6 to 8 weeks after the completion of the course of chemoradiotherapy and in the regular follow up period. Grades of Stomatitis/pharyngitis were determined based on the Radiation Therapy Oncology Group (RTOG) scoring criteria. CBC, LFT, and RFT were checked weekly. The grades of the hematologic toxicities were also determined based on the RTOG scoring criteria. A complete response was defined as complete resolution of all clinically evident symptoms of the disease and imaging studies. A partial response was defined as at least 50% reduction in the tumor size and not meeting the criteria for complete response, and finally, a less than 50% tumor regression was considered as no clinical response. Clinical response rates and toxicities were the primary and secondary end-points of the study. Clinical response rates and toxicities were compared between the variables (sex, age, primary sites, stage, and tumor grade) using the Chi-Square or Fisher’s Exact test when the cell expectation was less than six. P values less than 0.05 were considered significant. All statistical analyses were performed with SPSS software (Version 15.0, Chicago, IL, USA).

## Results

From April 2001 to November 2005, fifty patients with acceptable inclusion criteria were enrolled, four of them did not complete their treatment program and finally the study was closed with 46 patients. There were 31 men and 15 women and all of them had non-metastatic, locally advanced (T2-3 N2-3 or T4N0-3) head and neck carcinomas (Table 1). At the time of data analysis, follow up period was at least 24 months with a median of 36 months. There were 26% (12/46) none response, 50% (23/46) partial and 24% (11/46) complete response to treatment. Advanced T4 classification was associated with poorer response rate (p=0.042). The major (grade 3-4) treatment- related toxicities were myelosuppression (89%), stomatitis/pharyngitis (30%), anorexia (13%), emesis (11%), and diarrhea (7%) (Table 2). Neutropenia was the most common hematologic adverse effect, there was no treatment–related mortality, and most of the patients were treated in an outpatient setting with GCSF support and prophylactic antibiotic therapy (in some cases), but there were 3 patients (7%) who needed hospitalization due to febrile neutropenia. Among non-hematologic adverse effects, mucositis, especially in oropharyngeal region, was most common.

**Table 1:** Patients characteristics

** Variables **	** Response **				
	** No **	** Partial **	** Complete **	** P value **	** Total **
Sex					
Male	8	17	6		31
Female	4	6	5	0.529	15
Age					
≤60 years	7	12	7		26
>60 years	5	11	4	0.811	20
Primary site					
Maxillary sinus	0	2	1		3
Larynx	6	10	8		24
Buccal mucosa	2	3	0		5
Tongue	2	3	1		6
Parotid	2	5	1	0.768	8
T classification					
2	1	4	2		7
3	4	10	8		22
4	7	9	1	0.042	17
N classification					
0	3	5	0		8
1	0	2	2		4
2	3	7	6		16
3	6	9	3	0.303	18
Histological grade					
1	2	1	2		5
2	6	8	5		19
3	4	14	4	0.411	22
Total	12	23	11	-	-

**Table 2:** Acute adverse events

** Toxicities **	** Grade 0-2 (%) **	** Grade 3-4 (%) **
Gastro-intestinal		
Anorexia	40 (87)	6 (13)
Diarrhea	43 (93)	3 (7)
Emesis	41 (89)	5 (11)
Stomatitis/pharyngitis	32 (70)	14 (30)
Hematologic	5 (11)	41 (89)
Neutropenia	8 (17)	38 (83)
Thrombocytopenia	44 (96)	2 (4)
Febrile neutropenia	43 (93)	3 (7)
Anemia	39 (85)	7 (15)

## Discussion

Historically, before the taxane era, combination chemotherapy was associated with an increased response rate but not an improved median survival time relative to monotherapy in the treatment of recurrent head and neck carcinomas. The taxanes produce singleagent response rates that equaled or exceeded those with cisplatin and 5-fluorouracil.[15][16][17][18][19][20]

Like most tumors, locally advanced, non-metastatic head and neck carcinomas can be considered a systemic disease and thus, an active systemic treatment should be administered. However, locoregional control of locally advanced head and neck carcinomas is a main goal of treatment.[19] It is a significant indicator for quality of life in this group of patients.

In patients with locally advanced, nonmetastatic carcinomas of the head and neck region who are previously untreated, treatment with cisplatin-based combination chemotherapy will yield major response rates approximating 90%, with clinical complete response rates in the 30% range.[15] It is also apparent that docetaxel has significant single agent activity in locally advanced head and neck carcinomas, and that additional activity is seen when the taxane is used together with conventional best therapy consisting of cisplatin and 5-fluorouracil. In this study, the results showed good response rates for chemotherapy with the TPF regimen (as induction), and chemoradiotherapy with acceptable toxicities in locally advanced, non-metastatic head and neck carcinomas. These results were also seen in other studies with induction chemotherapy (the TPF regimen) and concurrent chemoradiation. [16][17] Remnar et al.[17] in a study with 358 patients compared the PF (cisplatin + 5-FU) and TPF (docetaxel + cisplatin + 5-FU) regimens as induction chemotherapy followed by radiotherapy. They reported 54% and 68% response rates to induction chemotherapy respectively. The median progression free survival rates were 8.2 and 11 months and median overall survival rates were 14.2 and 18.6 months with a significant p value, respectively. Similar results were also seen by Hitt et al.[21] in 382 patients that compared PF and PacPF (paclitaxel + PF) followed by chemoradiotherapy. A large multi-institutional phase II trial also demonstrated equivalent and favorable times to local and distant progression at 91% and 87%, respectively, by adding induction chemotherapy to an intensive chemoradiotherapy regimen.[18]

In another study, sequential therapy with induction chemotherapy followed by concurrent chemoradiotherapy in locally advanced, non-metastatic head and neck carcinomas suggested that it could increase a complete response. The complete response rate can predict the locoregional control. Therefore, induction chemotherapy followed by chemoradiotherapy can improve the outcome of locally advanced head and neck carcinomas.[19]

In our study, although the incidence of grade 3 and 4 neutropenia was high (89%), only 7% (3 of 46 patients) required hospitalization due to febrile neutropenia, the rate of this complication was higher than what is usually reported with the PF chemotherapy regimen, but the hospitalization rate is approximately similar to the reported rates in other studies.[17][21] It may be related to the number of patients in this study. However, it seems to be better to use prophylactic granulocyte colony stimulating factor (G-CSF) for avoiding severe neutropenia and related morbidity. According to available data, current treatment guidelines from the National Comprehensive Cancer Network (NCCN) indicate no role for induction chemotherapy prior to planned surgery and post-operative radiation, and a limited role only in selected settings prior to radiation. [22] However, with the incorporation of taxanes into induction regimens containing cisplatin and 5-FU, newer data suggest that the indications for induction chemotherapy may evolve in the near future.[23]

In summary, we concluded that the use of the TPF regimen as induction and adjuvant chemotherapy with concurrent radiotherapy and weekly cisplatin can be an effective treatment program in locally advanced, non-metastatic head and neck carcinomas with acceptable toxicities. These results should be reevaluated in larger phase III clinical trials.
